# H syndrome treated with Tocilizumab: two case reports and literature review

**DOI:** 10.3389/fimmu.2023.1061182

**Published:** 2023-08-11

**Authors:** Robin Jacquot, Maurine Jouret, Mathieu Gerfaud Valentin, Maël Richard, Yvan Jamilloux, Florent Rousset, Jean-François Emile, Julien Haroche, Lars Steinmüller, Franck Zekre, Alice Phan, Alexandre Belot, Pascal Seve

**Affiliations:** ^1^ Department of Internal Medicine, University Hospital Lyon Croix-Rousse, Claude Bernard University – Lyon 1, Lyon, France; ^2^ Pediatric Nephrology, Rheumatology, Dermatology Unit, Hospices Civils de Lyon, Lyon, France; ^3^ Department of Internal Medicine, Hospital d’Ardèche Nord, Annonay, France; ^4^ Department of Anatomopathology, University Hospital Ambroise-Paré, Simone Veil University – Paris, Paris, France; ^5^ Department of Internal Medicine, University Hospital Pitié-Salpêtrière, Sorbonne University - Paris, Paris, France

**Keywords:** H syndrome, SLC29A3, tocilizumab, monogenic autoinflammatory disease, hyperpigmentation, recurrent febrile attacks, lymphoproliferative syndrome

## Abstract

H syndrome is a rare autosomal recessive genetic disorder characterized by the following clinical features: cutaneous hyperpigmentation, hypertrichosis, hepatosplenomegaly, heart anomalies, hearing loss, hypogonadism, short stature, hallux valgus, hyperglycemia, fixed flexion contractures of the toe joints, and the proximal interphalangeal joints. In rare cases, autoinflammatory and lymphoproliferative manifestations have also been reported. This disorder is due to loss-of-function mutations in *SLC29A3* gene, which encode the equilibrative nucleoside transporter ENT3. This deficiency leads to abnormal function and proliferation of histiocytes. H syndrome is part of the R-group of histiocytosis. We report two different cases, one was diagnosed in adulthood and the other in childhood. The first case reported is a 37-year-old woman suffering from H syndrome with an autoinflammatory systemic disease that begins in adulthood (fever and diffuse organ’s infiltration) and with cutaneous, articular, auditory, and endocrinological manifestations since childhood. The second case reported is a 2-year-old girl with autoinflammatory, endocrine, and cutaneous symptoms (fever, lymphadenopathy, organomegaly, growth delay, and cutaneous hyperpigmentation). Homozygous mutations in *SLC29A3* confirmed the diagnosis of H syndrome in both cases. Each patient was treated with Tocilizumab with a significant improvement for lymphoproliferative, autoinflammatory, and cutaneous manifestations. Both cases were reported to show the multiple characteristics of this rare syndrome, which can be diagnosed either in childhood or in adulthood. In addition, an overview of the literature suggested Tocilizumab efficiency.

## 1 Introduction

H syndrome is an autosomal recessive disorder of abnormal histiocytic proliferation caused by mutations in *SLC29A3* at chromosome 10q22.1 encoding the human equilibrative nucleoside transporter-3 (hENT3) protein. ENT3 is a member of the SLC29-equilibrating nucleoside transporter family, which has a widespread tissue distribution, and its cellular expression is particularly important in lysosomal and mitochondria membrane (1). Impaired phagocytosis by *SLC29A3* mutations result in excessive inflammatory response and histiocytic proliferation underlying clinical features (2). By searching on PubMed Medline using the keywords, H syndrome and SLC29A3, approximately 130 patients suffering from H syndrome have been reported in the literature until October 2022.

The main clinical manifestations associate cutaneous and systemic features starting with the letter “H”: hyperpigmentation and hypertrichosis of the inner thighs, hepatosplenomegaly, heart anomalies, hearing loss, hypogonadism, and low height. Systemic features including recurrent fever, arthritis, joint deformity, diabetes, and symptoms associated with diffuse organ infiltration are also described (3).

Both the cases we report had inflammatory manifestations that positively responded with Tocilizumab. In addition, we provide an overview of the nine other cases of H syndrome treated with Tocilizumab in the literature.

## 2 Case description and diagnostic assessment

### 2.1 Case report 1

A 37-year-old Moroccan woman was referred to our internal medicine department, for recurrent febrile attacks with abdominal pain and assessment of a fibro-inflammatory disease with mediastinal, pericardial, pleural, periaortic, perirenal, and retroperitoneum infiltration. She reported a sensorineural hearing loss and symmetrical hyperpigmentation with thickening of the legs 20 years prior to the current presentation.

**Figure 1:**
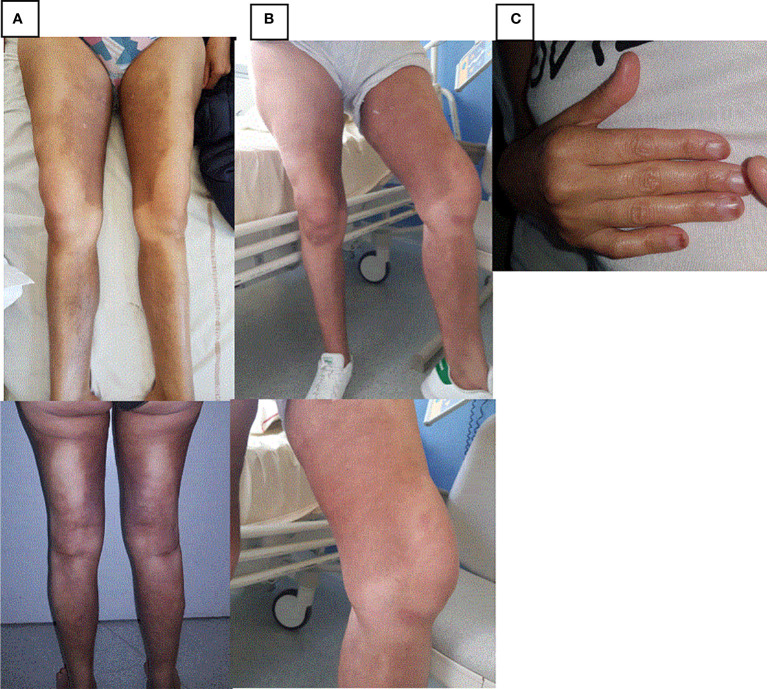
Skin lesions in case report 1. **(A)** Hyperpigmented and indurated cutaneous plaques inside both legs before treatment by Tocilizumab. **(B)** Hyperpigmented and indurated cutaneous plaques inside both legs 6 months after treatment by Tocilizumab. **(C)** Camptodactyly in the fifth radii.

When hospitalized, she complained of abdominal pain associated with nausea, vomiting, and fever. She reported tiredness, a weight loss of 8 kg, and night sweats for several weeks. On clinical examination, hyperpigmented and indurated cutaneous plaques inside both legs and sparing knees were found (**Figures 1A, B**). A camptodactyly in the fifth radii of both hands (**Figure 1C**) and hallux valgus and retraction of the toes were noticed. Laboratory test results revealed inflammation with C-reactive protein (CRP) at 97 mg/L (normal < 5 mg/L). There was no cytopenia. Two fasting blood glucose > 1.2 g/L led to diagnosis of diabetes with HbA1c concentration at 7.7%. An abdomino-pelvic computed tomography (CT) scan showed splenomegaly and retroperitoneal, periaortic, bilateral perirenal, pericardial, diffuse abdominal infiltration and right pleural effusion (**Table 1**).

**Table 1:** Clinical and biological response to Tocilizumab therapy.

	CASE 1		CASE 2		
	*Before Tocilizumab therapy*	*After 6 month of tocilizumab therapy (8 mg/kg) combined with corticosteroid*	*Before Tocilizumab therapy*	*After 3 months of* *treatment by* *Tocilizumab 10 mg/kg every 2 weeks*	*After 3 years treatment by* *Tocilizumab 12 mg/kg every 3 weeks*
Clinical features					
**Fever**	Yes	No (until 1 week)	Yes	Total improvement (until 3 months)	Total improvement
**Lymphadenopathy**	Yes	Regression	Yes	Partial improvement	Total improvement
**Splenomegaly**	Yes	Regression	Yes	Partial improvement	Total improvement
**Mediastinal lymphadenopathy**	Yes	Regression	–	–	–
**Cutaneous manifestations**	Yes	Partial improvement	Yes	Yes	Total improvement
**Heart infiltration**	Yes	Ineffective	–	–	
**Peri-renal infiltration**	Yes	Regression	–	–	–
**Peri-aortic infiltration**	Yes	Minimal improvement	–	–	–
**Fixed articular lesions** **(Hallux valgus, camptodactyly)**	Yes	Ineffective	–	–	–
**Deafness**	Yes	Ineffective	Yes	Ineffective	Ineffective
**Diabetes**	Yes	Ineffective	–	–	–
Laboratory tests					
**Inflammatory markers** **CRP** **SAA**			Constantly high 239 mg/L (N: < 5 mg/L) 44–234 mg/L (N: < 5 mg/L)	Normalized 2.4 mg/L (N: < 5 mg/L) <5 mg/L (N: < 5 mg/L)	Normal < 1 mg/L (N: < 1mg/L) < 5 mg/L (N: < 5 mg/L)

Investigations were conducted to exclude an infectious etiology (QuantiFERON^®^ test, syphilis, *Bartonella* sp., *Rickettsia* sp., and *Coxiella* sp. serology). There was no evidence to support the thesis of an autoimmune disease (negative antinuclear and antineutrophil cytoplasmic antibodies). Due to diffuse organ infiltration with inflammatory syndrome and a negative initial etiological assessment, non-Langheransian histiocytosis, IgG4-related disease, and lymphoma were considered.

Further studies showed no bone infiltration on bone scintigraphy and PET scan. Periaortic fatty infiltration associated with posterior mediastinal adenomegaly, paracardiac tumor lesions, and pericardial effusion on pan aortic MRI were observed. No pituitary gland infiltration on brain MRI was described. Right pleural effusion and thickening of the right atrium were found on thoracic CT scan, confirmed by cardiac ultrasound with a 5-cm tumor of the right atrium wall. Muscle MRI was done because of the infiltration of the inside thighs and showed subcutaneous soft tissue infiltration with edema, predominantly on the medial side of thighs with no muscle infiltration.

Skin biopsy and right video thoracoscopy with pleural and pericardial biopsy were performed as well. Skin histology revealed fibrosis hypodermic infiltrate histiocytes, CD 163+, CD 68+, CD1a−, PS100−, Langherine−, CD20− (no B cells), weakly positive IgG4 marking, with no BRAF/V600E mutation, no The mitogen-activated extracellular signal-regulated kinase (MEK)/The extracellular signal-regulated kinase (ERK) pathway mutation, and no immunohistochemical staining of phosphorylated ERK. Pleural and pericardial biopsy revealed the same histology.

The systemic manifestations (mediastinal, pleural, cardiac, retroperitoneum including kidney infiltration) and the histopathological characteristics (histiocyte’s infiltration with CD68+ and CD163+) aroused suspicions of histiocytosis. There was no pathognomonic long-bone osteosclerosis at the metadiaphysis to support the diagnosis of Erdheim–Chester disease. According to the patient’s clinical signs (cutaneous hyperpigmentation, camptodactyly with retraction of toes, diabetes mellitus, and history of sensorineural hearing loss), we suspected histiocytosis–lymphadenopathy plus syndrome, i.e., the H syndrome. The diagnosis was confirmed by genetic analysis using next-generation sequencing, which showed a homozygous mutation in the *SLCA29A3* gene c.1088G>A (p.Arg363Gln), which was performed thanks to Sanger sequencing.

Anti-IL6 therapy (Tocilizumab) was administered by intravenous injection (8 mg/kg/month) coupled with oral corticosteroid therapy (40 mg/day). Clinical features improved with a 8-kg gain, no more fever after 1 week, a decreased skin infiltration after 3 months, and the disappearance of splenomegaly. However, there was no result regarding deafness or joint damages. There was no inflammation on biological controls on the first month (**Table 1**). The reassessment CT scan on the 6 month showed a decrease in pericardial, mediastinal, perirenal, periaortic infiltration, and pleural effusion but no regression of atrium’s tumor.

### 2.2 Case report 2

The second case is a 2-year-old girl who was sent to our pediatric dermatology, nephrology, and rheumatology unit because of a hypertrichosis, hyperpigmentation, lymphoproliferative syndrome, and recurrent respiratory tract infections.

About her familial background, she was the offspring of consanguineous parents who comes from Algeria. She is the youngest daughter of a family with four children, two of whom already suffered from severe combined immune deficiency due to *RAG2* mutations.

**Figure 2:**
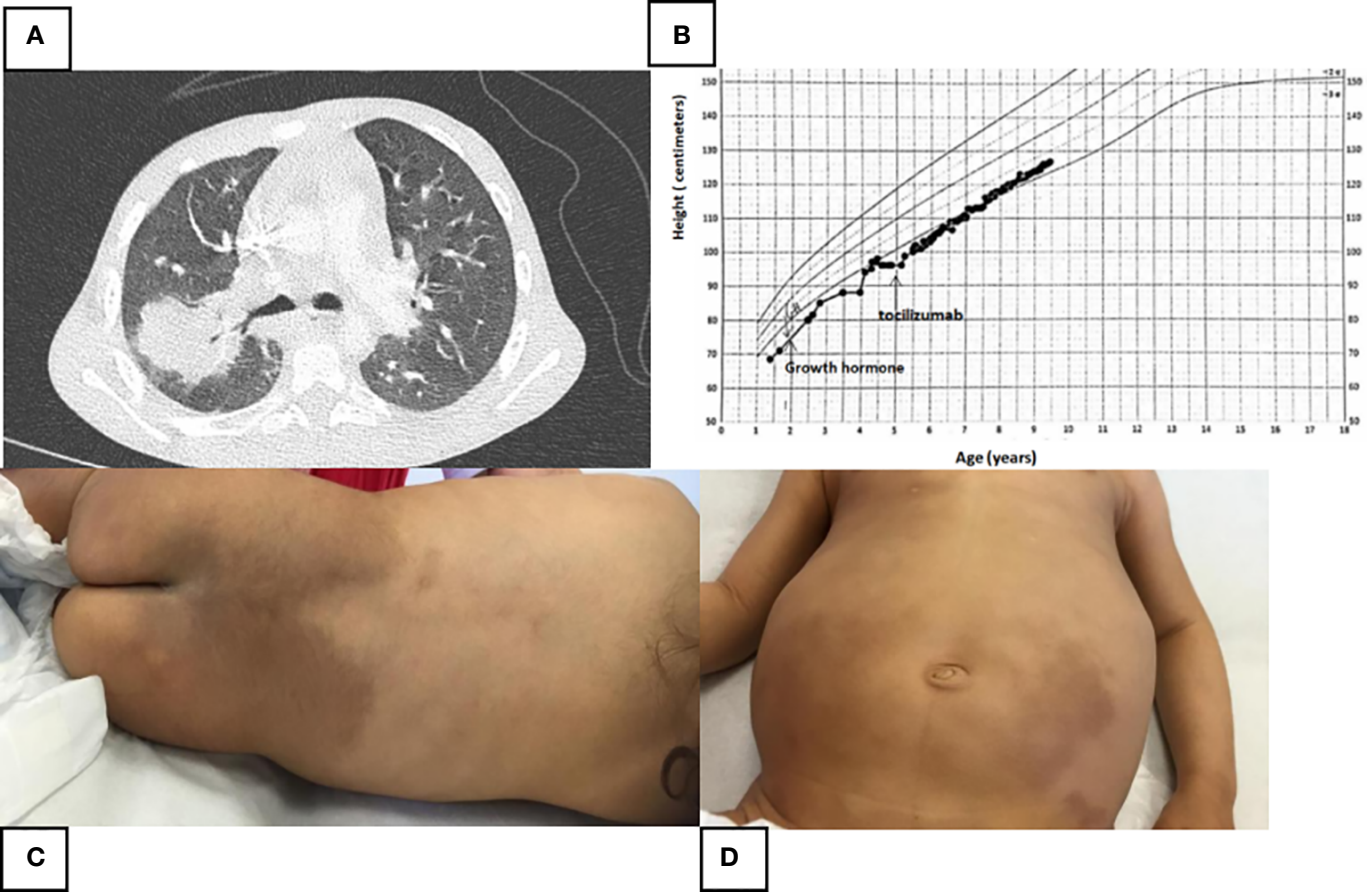
**(A)** Thoracic scanner (parenchymal window) performed at the age of 4 during a new feverish episode and respiratory difficulties. Acute postero-superior lobar pneumonia measuring 34 mm with air bronchogram, right hilar adenomegaly and sequelar mediobasal and posterobasal atelectasis. **(B)** Height growth chart. Moderate effect of growth hormone and Tocilizumab on the height. **(C, D)** Large areas of pigmentation of the lumbosacral region and both lower limbs with associated hypertrichosis.

From the first months of her life, she suffered from numerous febrile attacks and recurrent respiratory tract infections that required several hospitalizations (**Figure 2A**). Sensorineural hearing loss was identified at the age of 3. During the early childhood, she developed mild asthma with reactive airway disease well controlled with long acting beta2-agonist and montelukast. Since the age of 6 months, she presented a growth delay. The endocrine assessment revealed a delayed bone maturation and a partial growth hormone (GH) deficiency. MRI of the pituitary gland was normal. She has been supplemented with GH since the age of 18 months with moderate growth improvement (**Figure 2B**).

Her clinical examination revealed a significant hepatosplenomegaly. The most striking symptom was diffuse lymphadenopathy with many bilaterally cervical, axillary, and inguinal lymph nodes. Large areas of pigmentation of the lumbosacral region and of both the lower limbs with associated hypertrichosis were observed. The buttocks, knees, and popliteal fossae were spared (**Figures 2C, D**). There was a discrete lichenified appearance at the back of the feet. A delayed tooth eruption was noted. There was no skeletal deformities or facial dysmorphia. No abnormality was detected on cardiac, neurological, and genitourinary examination. Her psychomotor development was normal.

At admission, laboratory tests showed elevation of inflammatory markers including C-reactive protein at 239 mg/L (normal, <5 mg/L), serum amyloid A at 234 mg/L (normal < 5 mg/L), and erythrocyte sedimentation rate at 84 mm/h (normal < 21 mm/h). Interferon signature was moderately high at 6.6 (normal, 2.3). During febrile attacks, a microcytic hypochromic anemia was found (lowest measured hemoglobin level, 83 g/L; lowest Mean corpuscular volume (MCV), 61.7 fL). There were no autoantibodies found that suggested autoimmune disease. The blood glucose level was normal. Bone marrow aspiration was performed due to severe anemia and revealed a cell-rich bone marrow with balanced hematopoiesis. Lymphadenopathy biopsy showed reactive lymphadenitis with no specific feature of histiocytosis or H syndrome. No histiocytes and no sign of emperipolesis were found.

First genetic screening excluded the contribution of *RAG2*; the patient did not carry the familial mutation. Genetic tests by Sanger sequencing were conducted and revealed a homozygous mutation p.Arg363Trp in exon 6 of the *SLC29A3* gene. Hence, the diagnosis of H syndrome was confirmed. Regarding treatments, in the absence of an etiological diagnosis on the onset, Azathioprine (2 mg/kg/day) was introduced at the age of 3. Inflammatory markers remained elevated during treatment. No clinical improvement has been noted. We found the pathogenic mutation in *SLC29A3* at the age of 5, and consequently, the Tocilizumab was introduced at the dose of 10 mg/kg every 2 weeks. Inflammatory markers normalized 3 months after the beginning of the treatment. The patient suffers from repeated bronchopneumopathy, requiring intravenous antibiotics during the first months of the treatment. To prevent this side effect, the intake of Tocilizumab was decreased to every 3 weeks with the same dose (10 mg/kg). This protocol helped in fighting the infections but caused a resurgence of the lymphoproliferative disease with elevated inflammatory factors and clinical relapse (splenomegaly). An new dosage of Tocilizumab (12 mg/kg every 3 weeks) and a preventive treatment with alternate antibiotics (amoxicillin, sulfamethoxazol/trimetroprim, and clarithromycin) was administrated.

After 2 years of treatment with this amount, when the patient turned 7, a remission without cutaneous, lymphoproliferative, and inflammatory signs was observed. However, it did not have any notable effect upon deafness (**Table 1**).

Immunological assessment was performed to search for immune deficiency that could explain repeated lung infections. There was no leukopenia and no thrombocytopenia. T- and B-cell immunophenotyping, vaccine responses, and T lymphocyte functional test (OKT3 and PHA) were normal before the Tocilizumab was started. Importantly, T-cell subsets were normal without abnormal naive T cells and no bias in the immune repertoire. Hypergammaglobulinemia with IgG levels at 12.9 g/L (normal between 4.82 and 8.96 g/L) and IgM levels at 1.72 g/L (normal between 0.54 and 1.53 g/L) was noted. IgA level was normal for the age. These biological results invalidated the diagnosis of primary immune deficiency. After 3 years of treatment with Tocilizumab, at the age of 8, B-cell, T-cell, and NK-cell lymphopenia was observed to be associated with low rate of IgG2 and IgG4.

## 3 Discussion

### 3.1 Clinical features and differential diagnosis

In 2008, Molho-Pessach et al. first described H syndrome and reported mutation of *SLC29A3* as the cause of this syndrome (3). The majority of patients with H syndrome are of Arab origin especially because the two most common mutations were found in individuals of Arab–Palestinian ancestry (1). Since the first description was made, reported H syndrome cases demonstrated considerable clinical variability, which leads to misdiagnosis. This heterogeneity in phenotype may be attributed to impaired tissue response to injury (4).

H syndrome is considered as a familial Rosai–Dorfman–Destombes (RDD) disease, classified as R-group in revised classification of histiocytosis (5, 6). It is an autosomal recessive disorder, which is mainly diagnosed among children thanks to early symptom developments and family history. Typical clinical manifestations are hyperpigmentation (91%) and hypertrichosis (68%) of the inner thighs, flexion contractures of proximal interphalangeal joints (56%), short stature (49%), diabetes, hepatosplenomegaly (40%), and hallux valgus (25%) (7). Diffuse lymphadenopathy may occur in 24% (7) of cases and can mimicking RDD like in our pediatric case (8). In addition, this syndrome is part of the spectrum of monogenic autoinflammatory diseases (9) with 25% of patients developing autoinflammatory complications like recurrent febrile attacks, persistently elevated acute phase reactants, or fibrosis. The quality of tissue repair can be affected, causing fibrosis and histiocytic invasion of organs (10). Symptoms may be related to this diffuse tissue infiltration (pericardial, perirenal, periaortic, and retroperitoneal) as demonstrated on our adult case. Depending on how the organs are affected, it may be confused with other types of histiocytosis. By perirenal infiltration and right atrial tumor, our adult case was initially presented as Erdheim–Chester disease (ECD) clinical phenotype, but the absence of bone infiltration has challenged the diagnosis.

H syndrome being held responsible for specific cutaneous symptoms, it should induce such a diagnosis, and it may be associated with autoinflammatory manifestations and lymphadenopathy. However, these aspects require particular attention not to miss differential diagnosis of lymphoproliferative syndromes [Castleman disease, autoimmune lymphoproliferative syndrome (ALPS) and non-Langerhans histiocytosis such as RDD and ECD] and genetic autoinflammatory diseases (mevalonate kinase deficiency) (11, 12).

### 3.2 Histology

The key element to diagnose this syndrome is a histological analysis of lymph nodes and/or infiltration tissues. Cutaneous and organ infiltration biopsy normally showed a dense dermal and subcutaneous infiltrate, composed of CD68+ histiocytes and later replaced by fibrosis (13). In our first case, the patient presented mediastinal, pericardial, pleural, periaortic, perirenal, and retroperitoneal diffuse infiltrations, which suggest clinical characteristics of ECD, but without bones involvement. Histopathological characteristics (CD 163+, CD 68+, CD1a−, PS100−, Langherine−) exclude the diagnosis of Langerhans histiocytosis and brings us to an ECD rather than an RDD because of lack of S-100 protein labeling. These clinical and histological immunophenotypes need to be considered because, traditionally, H syndrome phenotype is closer to RDD’s manifestations with S-100 protein positivity and no emperipolesis (7, 13). Therefore, histological analysis usually leads to a diagnosis of histiocytosis, with markers that rule our Langerhans histiocytosis but can be consistent with ECD or RDD histology. Histological analysis is essential to determine the proper diagnosis.

However, histological analyses have limits; as shown in our second case, reactive lymphadenitis were observed without specific microscopic features of histiocytosis. Biopsy of lymph node or infiltration tissues may help clinicians with histopathological characteristics (CD163, CD68, CD1a, PS100, and Langherine); however, there is no specific feature of syndrome H (13).

### 3.3 Genetic

Genetic testing was performed after the informed consent provision revealed a homozygous mutation c.1088G>A p.Arg363Gln in exon 6 in senior patient report and c.1087C>T p.Arg363Trp in exon 6 of the *SLC29A3* gene in pediatric patient report. The mutations highlighted within our studies have already been described among patients with H syndrome (14, 15). The residue 363 encodes for a highly conserved position in the protein. c.1087C>T p.Arg363Trp is predicted to be likely pathogenic by the browser Polyphen and is rare in the general population (allele frequency in gnomAD: q =0.00004773, <0.0001 is the threshold for recessive gene). c.1088G>A is predicted to be pathogenic by Polyphen and its low-frequency variant (q = 0.000007955).

In our pediatric case, it was found that both parents were heterozygous for this mutation. A highly conserved position in the protein, segregation of this mutation in the family members, and low frequency of the variant in the general population supported its pathogenicity.

### 3.4 Treatment and review of the literature

Regarding the complexity of H syndrome, there is a lack of consensus about the treatment. Colchicine, anti-IL1, and Tumor necrosis factor (TNF)-alpha therapy seem to be ineffective (15). Systemic corticosteroids showed good results but with important harmful long-term effect, especially among diabetic patients diagnosed as H syndrome (16). Some of these patients partially responded to a methotrexate treatment followed by a notable improvement on hyperpigmentation, joint stiffness, and arthritis (17). Methotrexate can be combined with Tocilizumab or Ciclosporine to keep the disease under control (9, 18) (**Table 2**). Generally, Ciclosporine receded the symptoms, but arthritis and persistent inflammation did not improve with the mentioned treatment (9). The positive effect on mycophenolate mofetil is also described on hyperpigmentation and joint stiffness with promising results (24). What seems to be the advantage of Tocilizumab is the fact that this medication has higher success in the treatment of inflammatory manifestations such as arthritis and organ infiltration (16).

**Table 2:** Characteristics of H syndrome treated by Tocilizumab.

Patient	Age (years) Gender	Mutation	Effective dose of Tocilizumab	Improvements	No response	Side effects	Reference
**Patient 1**	21 Female	Homozygous c.300 + 1G>A	Unspecified	Normalizing CRP	No clinical improvement on cutaneous and musculoskeletal symptoms (arthralgia, myalgia, skeletal abnormalities)	Not reported	Mistry, 2016(19)
**Patient 2**	12 Female	Homozygous c.1346C>G	12 mg/kg every 2 weeks Initially started with methotrexate and steroids then used alone	Improvement of the general condition, decrease in febrile episode and night sweats, subjectively less skin tightness	–	4 respiratory tract infections requiring intravenous antibiotics	Rafiq, 2017 (18)
**Patient 3**	19 Male	Homozygous c.1087C > T	12 mg/kg/2 weeks IV	Normalizing CRP and ESR Partial clinical response on arthritis (wrists, knees, ankles, fingers)	–	Not reported	Bloom, 2017 (9)
**Patient 4**	16 Male	Homozygous c.347 T > G c.610 + 1G>C	10 mg/kg/2 weeks IV then switched to Tocilizumab 162 mg twice a week SC	Improvement in symptoms and inflammatory markers Resolving microcytic anemia, thrombocytosis Thickened skin normalized Increase in growth velocity (in association of growth hormone)	Persistant hirsutism, dyslipidemia IgG2 and IgG4 subclass deficiency	Not reported	Bloom, 2017 (9)
**Patient 5**	16 Female	Homozygous c.1309G>A	162 mg every week SC in combinaison with methotrexate 25 mg per weeks	Improvement of the skin lesions including skin tone and shrinking in size	–	Not reported	Bloom, 2017 (9)
**Patient 6**	34 Female	Unspecified	8 mg/kg/2 weeks IV	Improvement of skin lesions (hyperpigmentation and infiltration)	–	Not reported	Hamann, 2019 (20)
**Patient 7**	8 Male	Homozygous c.971C > T	8 mg/kg/2 weeks IV	Improvement of the multiorgan infiltration with impressive clinical improvement on heart, lung, liver and kidney failure	–	Not reported	Ventura, 2021 (21)
**Patient 8**	Tolder Male	Unspecified	Unspecified	Improvement of inflammatory markers, sensorineural hearing loss was notable for improvement and stabilization	–	Not reported	Gunderman, 2022 (22)
**Patient 9**	6 Male	Compound heterozygous c.1157G>A and c.890dupC	8 mg/kg/2weeks during one month	–	No improvement on pure red cell anemia	Hepatotoxicity after 2 perfusions (increased AST/ALT 2 x normal value) Normalization after discontinuation of treatment	Besci, 2022 (23)
**Patient 10**	5 Female	Homozygous c.1087C>T	12 mg/kg/3 weeks	Disappearance of hypertrichosis and hyperpigmentation, lymphoproliferation, recurrent fever, inflammatory markers and microcytic anemia	Sensorineural hearing loss continued to rise moderately	Respiratory tract infection needed antibiotic treatments.	Case described in this article
**Patient 11**	37 Female	Homozygous c.1088G>A	8 mg/kg/4 weeks	Decreased in skin, pericardial, perirenal, retroperitoneal infiltration, resolution of fever, improvement of the general status and reduction of biological inflammation	Persistence of heart infiltration and partial improvement of peri-renal and peri-aortic infiltration	–	Case described in this article

IV, intravenous; SC, subcutaneous.

In the first case’s treatment, Tocilizumab (8 mg/kg/4 weeks) was the better option to start with. It improved the hyperpigmentation and pericardial, perirenal, and retroperitoneal infiltration; broke down the fever; improved the general status; and reduced the inflammation (**Table 2**). However, despite the use of Tocilizumab, a 5-cm right atrial infiltration remained as it was initially, which led to changing the treatment. No side effect was noticed.

In the second case, Tocilizumab happened to be effective when increased (10 mg/kg per 2 weeks). However, it needed to be balanced because of lower respiratory tract infections. A higher dose of 12 mg/kg/3 week, paired with preventive antibiotic treatment, proved to more effective. Typical posology for systemic juvenile idiopathic arthritis (S-JIA) approved by Food and Drug Administration (FDA) regarding children weighing <30 kg is 12 mg/kg every 2 weeks. The most common adverse effect is infections (mild gastroenteritis and nasopharyngeal and upper respiratory infections) along with biological abnormalities (neutropenia, thrombopenia, lymphopenia, and elevated transaminases) (25). In safety studies of Tocilizumab in patients with systemic-onset juvenile idiopathic arthritis, cases of bronchopneumonia are rare (n=4/112) and are treated with antibiotics (26).

To stress the efficiency of Tocilizumab, we can say that among the 134 H-syndrome-confirmed patients reviewed in the literature, there were 11 patients (including our cases) with pathogenic *SCL29A3* mutations treated with Tocilizumab (**Table 2**).

The median age of the patients was 14 years (extreme values, 5–37 years). Doses used varied between 8 mg/kg/4 weeks and 12 mg/kg/2 weeks. Patients 1 and 9 did not respond to treatment with persistent cutaneous manifestations, arthralgia and skeletal abnormalities, and pure red cell aplasia. For one patient (our adult case), Tocilizumab was discontinued because of a partial response on tissue infiltration with Tocilizumab 8 mg/kg/4weeks. The other eight patients had notably responded to Tocilizumab. Improvement was described regarding cutaneous symptoms (5/6), arthritis (1 with partial response), microcytic anemia (2/2), recurrent fever (3/3), lymphoproliferation (1/1), growth velocity (2/2, adding to growth hormones), hearing loss (1/2), acute phase reactants (5/5), and multiorgan infiltration (2/2). Two patients benefited from subcutaneous Tocilizumab injections (162 mg every 2 weeks) with successful response. Regarding the side effects, two patients presented recurrent tract infections, which were checked, thanks to a lower dosage but no crucial need to discontinue the treatment. Only one patient presented a moderate hepatotoxicity (moderate elevation AST/ALT, 1/11), which required stopping Tocilizumab use.

Even though Tocilizumab seems to be effective for these patients, the data from this study must be interpreted according to the following limitations: retrospective data, small number of patients included, and lack of comparative information with other treatments.

## 4 Patient perspective

In the first case, persistence of heart infiltration and partial improvement of peri-renal and peri-aortic infiltration, anti-MEK (COBIMETINIB) has been discussed in a multidisciplinary meeting, despite the absence of ERK hyperphosphorylation on biopsy.

Concerning our pediatric patient, she is currently 9 years old and has been treated with Tocilizumab for 4 years with a 12 mg/kg/3 weeks dose. Until now, no lymphoproliferation or clinical or biological inflammation was reported, and skin lesions have disappeared. Growth velocity has been improved thanks to the intake of growth hormone and the control of systemic inflammation with Tocilizumab, but sensorineural hearing loss continues to evolve moderately and slowly.

## 5 Conclusion

This report shows that H syndrome, due to mutation in *SLC29A3*, leads to complex clinical manifestations, which can be diagnosed in adulthood with a fibro-inflammatory disease-like presentation and in childhood with lymphoproliferative syndrome associated with inflammatory condition. We suggest that Tocilizumab should be considered as the first-choice treatment, mainly to control autoinflammatory component (skin lesions, lymphoproliferation, recurrent fever, arthritis, and systemic fibrosis). The treatment intensification with doses increased to 12 mg/kg/2 weeks may be considered when the clinical response is partial, prior to a change in treatment. Tocilizumab treatment may be taken as early as possible, ideally as a juvenile to avoid chronic inflammatory complications.

## Data availability statement

The original contributions presented in the study are included in the article/supplementary material. Further inquiries can be directed to the corresponding author.

## Ethics statement

Written informed consent was obtained from the individual(s), and minor(s)’ legal guardian/next of kin, for the publication of any potentially identifiable images or data included in this article.

## Author contributions

All authors listed have made a substantial, direct, and intellectual contribution to the work and approved it for publication.
